# Spontaneous Coronary Artery Dissection in an Elderly Postmenopausal Nigerian Woman With Recurrent Chest Pain: A Case Report

**DOI:** 10.7759/cureus.100812

**Published:** 2026-01-05

**Authors:** Olurotimi J Badero, Oyewole Kushimo, Bamikole Osibowale, Victor Ajayi, Adaobi Ofordile

**Affiliations:** 1 Interventional Cardiology, Iwosan Lagoon Hospital, Lagos, NGA; 2 Interventional Cardiology, Division of Cardio-Nephrology, Cardiac Renal and Vascular Associates PC, Jackson, USA; 3 Internal Medicine, Lagos University Teaching Hospital, Lagos, NGA; 4 Cardiology, Caribbean Tristate Heart Institute, Trinidad, TTO; 5 Medicine, Lagos State University Teaching Hospital, Lagos, NGA; 6 Medicine/Cardiology, Lagos University Teaching Hospital, Lagos, NGA

**Keywords:** chest pain, coronary artery disease, coronary artery dissection, nigeria, postmenopausal

## Abstract

Spontaneous coronary artery dissection (SCAD) is an unusual etiology of myocardial infarction and sudden cardiac death (SCD) with a different pathophysiological mechanism. Often associated with younger women, where the hormonal milieu of estrogen and progesterone has been implicated, postmenopausal SCAD is, however, uncommon with unknown management outcomes.

We report the case of a 75-year-old postmenopausal woman presenting with recurrent chest pain, found to have SCAD on coronary angiography, and managed conservatively.

We believe this represents one of the earliest documented cases of postmenopausal SCAD in Nigeria diagnosed angiographically. It seeks to enhance local awareness, review current diagnostic and management modalities, and contribute to the expanding body of literature.

SCAD can present as acute coronary syndrome (ACS) in young women, where the hormonal milieu has been implicated. Postmenopausal SCAD is, however, uncommon, and a high index of suspicion is essential to prevent adverse outcomes.

## Introduction

Spontaneous coronary artery dissection, a previously presumed rare entity, is now being recognized as an important etiology of acute coronary syndrome (ACS) and sudden cardiac death (SCD) among younger women [1]. It is characterized by a spontaneous, non-atherosclerotic, non-traumatic, and non-iatrogenic separation of the coronary artery wall by an intramural hematoma, which may or may not be associated with an intimal tear. The accumulation of blood within the arterial wall can compress the true lumen, reducing coronary blood flow and potentially leading to myocardial ischemia or infarction [2].

SCAD predominantly affects younger premenopausal women, with the mean age of diagnosis spanning 44-55 years [3]; hence, SCAD in an older postmenopausal woman is unusual. The overall prevalence of SCAD is reported to be about 4% of all ACS and is responsible for about 35% of ACS seen in women younger than 50 years [4].

The clinical presentation of SCAD mirrors that of atherosclerotic plaque rupture-induced ACS, typically manifesting as classic chest pain accompanied by diagnostic ECG abnormalities and elevated cardiac biomarkers. However, traditional cardiovascular risks are usually absent. The diagnosis of SCAD has been enhanced by advanced imaging techniques, including optical coherence tomography (OCT) and intravascular ultrasound (IVUS), especially in patients with unusual angiographic patterns. However, their availability remains limited in developing countries.

Older patients with SCAD exhibit a distinct clinical and angiographic phenotype compared to their younger counterparts, with different initial treatment strategies, but similar in-hospital outcomes [4].

We present a 75-year-old Nigerian woman with hypertension, diabetes, and chronic kidney disease (CKD) who was diagnosed with SCAD. This case highlights the need for a high index of suspicion among older women presenting with ACS.

## Case presentation

A 75-year-old woman with past medical history significant for type 2 diabetes mellitus, hypertension, and chronic kidney disease (CKD) stage 4 with an estimated glomerular filtration rate (eGFR) of 25 cc/minute per chronic kidney disease-epidemiology collaboration (CKD-EPI), was evaluated at the cardiology clinic for chest pain. The chest pain was described as stabbing in nature, mid-sternal in location, exertional, and associated with nausea and shortness of breath. The symptoms had progressively worsened in frequency and severity over the previous weeks, with symptoms now occurring at rest.

**Figure 1:**
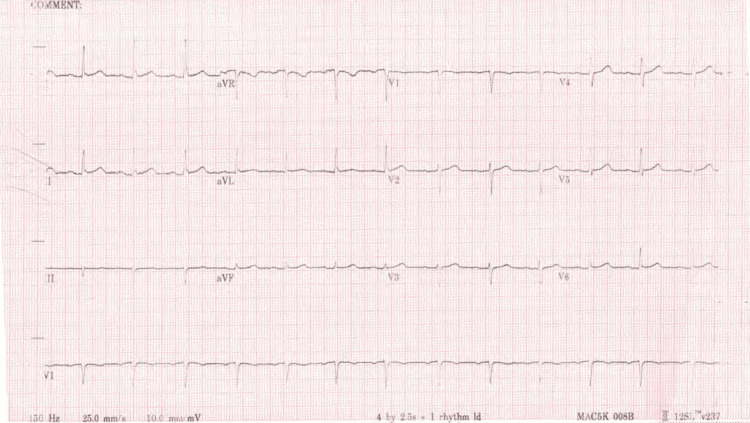
Electrocardiographic image showing normal sinus rhythm

**Figure 2:**
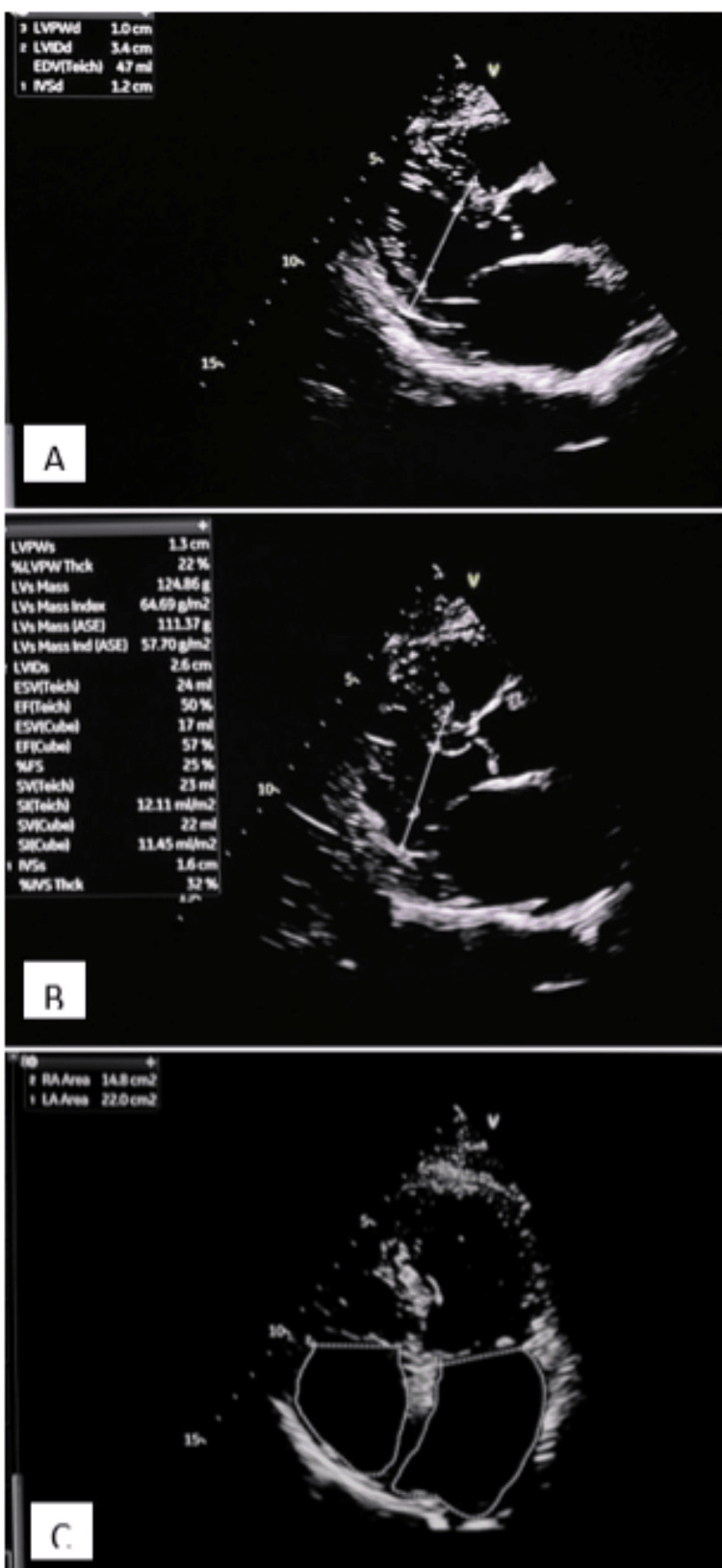
Echocardiographic images showing concentric left ventricular hypertrophy and dilated left atrium A: Parasternal long-axis view in systole, with EF of 50%. B: Parasternal long-axis view in diastole; the interventricular septal wall thickness in diastole measures 1.2 cm. C: Apical four-chamber view showing dilated left atrium; the left atrial area measures 22 cm^2^. EF: ejection fraction

An electrocardiogram showed a normal sinus rhythm with no acute ST-T wave changes (Figure 1). Echocardiography demonstrated left ventricular (LV) concentric hypertrophy with a low normal LV ejection fraction of 50% and a dilated left atrium (Figure 2). There was no significant valvular disease. A diagnosis of unstable angina was made with a thrombolysis in myocardial infarction (TIMI) risk score of 3. She was planned for an early invasive strategy with a coronary angiogram.

**Figure 3:**
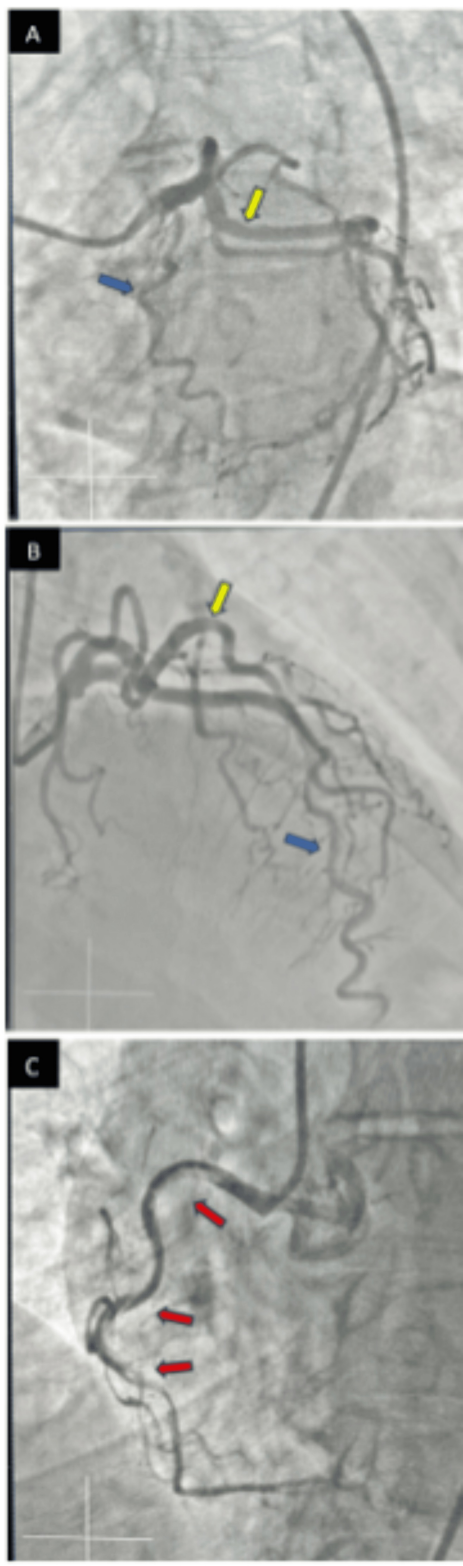
Angiographic images A: Coronary angiogram showing left anterior descending (blue arrow) and left circumflex (yellow arrow). B: Coronary angiogram showing left anterior descending (blue arrow) and left circumflex (yellow arrow). C: Coronary angiogram showing right coronary artery with spontaneous coronary artery dissection (red arrows).

Coronary angiography in multiple orthogonal views was performed the next day under moderate sedation via right common femoral access using JL-4 and JR-4 diagnostic catheters. Coronary angiogram findings revealed angiographically normal left main, tortuous left anterior descending artery with mild luminal irregularities, and angiographically normal left circumflex artery (Figure 3A and Figure 3B). The right coronary artery showed a type 1 SCAD involving the proximal, mid, and distal segments of the artery (Figure 3C). She was managed medically with aspirin and beta-blockers with close follow-up.

Our patient has been doing well on follow-up but continues to experience intermittent episodes of chest pain with decreased frequency and intensity from the initial presentation. She has not reported any chest pain at rest. She is being continued on beta-blockers and aspirin with risk factor and lifestyle modification.

## Discussion

SCAD is a non-traumatic and non-iatrogenic coronary event that involves the formation of a hematoma within the tunica media complex, resulting in the splitting of the innermost layer of the blood vessel wall (intima) from the rest of the wall, forming a flap that squeezes the main channel and restricts blood flow, causing ischemia or a myocardial infarction [3,5]. SCAD is reported more commonly in the cohort of premenopausal women with an ACS. A case of SCAD has been reported in a premenopausal Nigerian woman with pregnancy-associated acute myocardial infarction [6]. We, however, report the first case of SCAD in an elderly postmenopausal woman in Nigeria [6]. The occurrence of SCAD in postmenopausal women highlights the multifactorial nature of the conditions and the need to consider other non-hormonal factors, such as connective tissue disorders (fibromuscular dysplasia, Marfan’s syndrome, and Ehlers-Danlos syndrome), and genetic predisposition [3,5].

Some differences in SCAD characteristics have been identified in postmenopausal women. A Spanish multicenter registry noted that postmenopausal women had a previous history of acute coronary syndrome more often but presented less frequently as ST-segment elevation myocardial infarction on admission and less frequently with left ventricular dysfunction compared with premenopausal women [7]. These characteristics were noted in our index patient, who presented with unstable angina and preserved LV systolic function. Older patients were also observed to have hypertension and dyslipidemia, with an identifiable trigger less often present. They also more often had severe coronary tortuosity (like our patient) and coronary ectasia. There was no significant difference in major adverse cardiac events, heart failure, or in-hospital stay during the index admission compared to younger patients [7].

The diagnosis of SCAD is often made by invasive coronary angiography. Additional intravascular imaging with OCT or IVUS is reserved for cases of diagnostic uncertainty or when percutaneous coronary intervention (PCI) is anticipated. Most cases of SCAD are managed conservatively in view of the high probability of spontaneous healing and the high risks of revascularization. A study that analyzed patients with SCAD who had a subsequent coronary angiography for diverse indications showed that 95% had angiographic healing 30 days post-event [8]. In as many as one-third of PCI procedures, the intramural hematoma propagates frequently, necessitating the use of several unplanned stents [9]. As the hematoma resorbs, it may lead to late strut misalignment [3].

Conservative management, especially in the acute phase, can lead to complete healing and is usually recommended, as percutaneous coronary intervention (PCI) is associated with high failure rates and iatrogenic dissections. We adopted a conservative approach in our patient, given preserved vessel flow and the absence of ongoing ischemia.

## Conclusions

Spontaneous coronary artery dissection (SCAD) remains an infrequent, elusive, and challenging clinical entity of multifactorial etiology, many years after it was first described. Intravascular imaging may be necessary when the diagnosis is uncertain, and a conservative approach has been shown to give the best outcomes. Increased awareness of this condition, particularly in developing countries, is crucial, and more research needs to be done to further understand this entity and add to the growing body of literature.
